# Itch in Scabies—What Do We Know?

**DOI:** 10.3389/fmed.2021.628392

**Published:** 2021-02-01

**Authors:** Sascha Ständer, Sonja Ständer

**Affiliations:** ^1^Department of Dermatology, University of Lübeck, Lübeck, Germany; ^2^Department of Dermatology, Center for Chronic Pruritus, University Hospital Münster, Münster, Germany

**Keywords:** scabies, itch, pruritus, itch mediators, itch and its pathways

## Abstract

Scabies is a common parasitic skin infestation characterized by severe itch and a heterogenous clinical presentation. Itch, as the cardinal symptom of scabies, is imposing a high burden on affected patients and is often difficult to manage. Decreased life quality and secondary complications, caused by an itch-related disruption of the epidermal barrier and subsequent superinfections, illustrate the need to treat scabies and to understand the underlying mechanisms of itch in respective patients. This review summarizes available data on itch in scabies with a special focus on the clinical aspects and its underlying pathomechanisms.

## Scabies—An Overview

Scabies is a highly contagious ectoparasitic skin infestation caused by Sarcoptes scabiei var. hominis. With a global prevalence of 204 million, scabies poses a remarkable burden on both infected individuals and on the healthcare system regardless of the socioeconomic standard of the respective country (1, 2). More specifically, in high income countries, delayed diagnosis of this neglected disease can lead to waves of institutional outbreaks. In middle-to-low income countries a lack of therapeutic resources often results in secondary scabies-related complications, such as chronic kidney disease since excoriations and the disruption of the epidermal barrier caused by itch may lead to impetigo and to a subsequent dissemination of streptococci to the glomeruli (1, 3). The increased disease-related morbidity and mortality further adds to the patients' burden. Depending on different populations, the prevalence of scabies is ranging from 0.2 to 71.4% (4), with a predominant affection of people living in tropical regions (5). While scabies can occur in every individual, current data reveal a greater susceptibility for the young, old, and, generally, immunocompromised patients (6). Accordingly, Mason et al. reported the highest prevalence of scabies in infants <1 year of age [34.1%, adjusted odds ratio (AOR) compared with adults: 3.6, 95%CI 2.2–6.0] and children aged 1–4 years (25.7%, AOR 2.6, 95%CI 1.7–3.9) in the Solomon Islands (7). Furthermore, the disability-adjusted life-years (DALY) burden was found to be the highest in children 1–4 years-old, eventually decreasing from age 5–24 years and recurrently rising after the age of 70 years in the Global Burden of Disease Study 2015 (5). Furthermore, in low-income countries, disadvantaged populations and children under the age of two bare a greater risk to get infected with scabies (4).

The mite Sarcoptes scabiei is an obligate human parasite that burrows into the epidermis mostly after intense skin to skin contact but also after contact to mites from textiles (i.e., sleeping in a bed with mites). The female mite lays eggs and after approximately 14 days the hatched larvae, and later the nymph, reach adulthood (8), provoking symptoms mostly after 2-5 weeks of latency after the first infestation. After a second infestation, the aforementioned symptoms can occur earlier (~ after 1–2 weeks) due to the immune memory. As scabies mites prefer areas with a higher body temperature and a rather thin stratum corneum, predilection sites are the interdigital spaces of hands and feet, the axillary and periumbilical region, the penis and the perianal skin, while the head and neck are usually spared (exception in infants and old people) (9).

**Figure 1:**
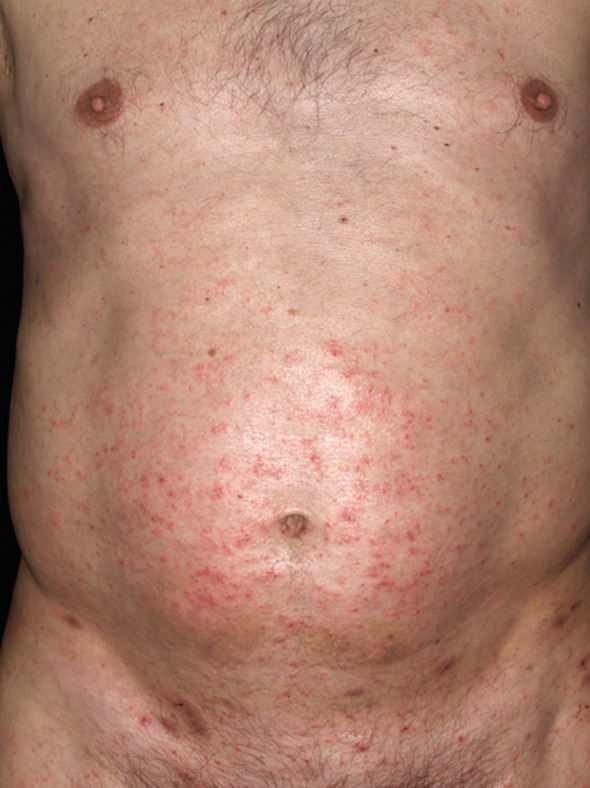
Clinical presentation of a male patient infected with scabies: Multiple erythematous papules in the periumbilical region with several scratch lesions and excoriations.

The clinical picture of the disease is caused by the infestation of the mites *per se* and by an immunologic reaction elicited by the contact toward the by-products of the mites (i.e., saliva, excrements). This delayed type reaction of cellular immunity is clinically apparent as an eczematous morphology with multiple disseminated erythematous papules and vesicles on red skin (Figure 1). Due to an intense pruritus, especially at night, secondary skin lesions, that is, excoriations and scratch marks occur frequently. Secondary superinfections in terms of impetigo can frequently be observed in children.

**Figure 2:**
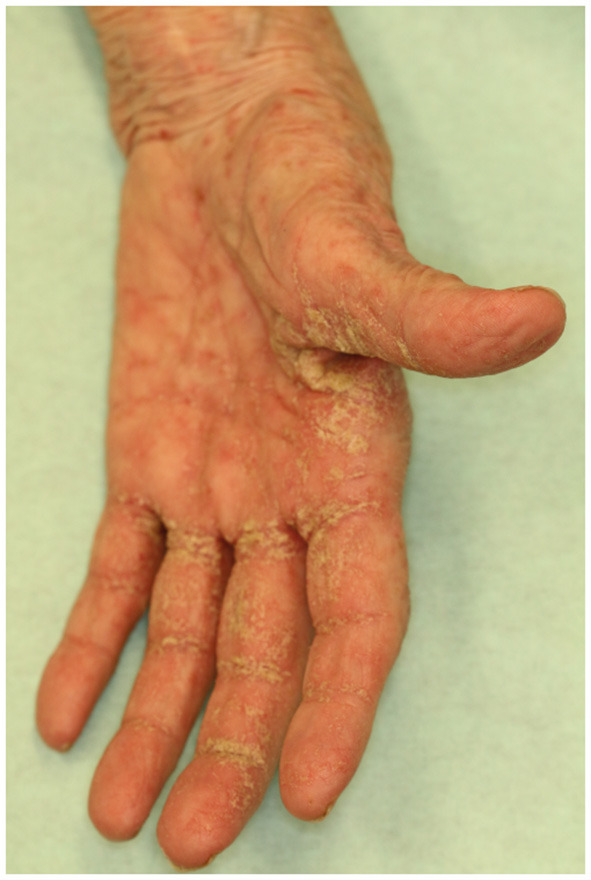
Crusted scabies: Interdigitally located squamous plaques and disseminated erythematous papules.

Scabies crustosa (norvegica), a comparably rare and severe form of scabies with a prevalence of <0.1%, is especially apparent in individuals with an underlying immunosuppression, that is, HIV (10). The localized or generalized hyperkeratotic clinical picture results from a massive mite proliferation and is associated with an increased mortality and a lower grade of itch (10, 11) (Figure 2).

Diagnosis of scabies is based on a characteristic history (intense pruritus with a nightly deterioration, frequent relapses and refractory to treatment with topical corticosteroids) and the clinical presentation with skin lesions along the predilection sites and typical findings in dermatoscopy and positive microscopy, that is, mites, feces, eggs or mite passages.

Currently, topical treatment with permethrin cream (5%) is considered as the gold standard, leading to a cure in most of the cases (12). Yet, increasing numbers of failures in treatment by permethrin have been recently reported also hinting toward the possibility of increased tolerance of permethrin (when applied under controlled conditions) or possible application errors (13, 14). Successful treatment, however, highly depends on the appropriate application of the cream (whole body from the neck) and concomitant treatment of persons with close contact and basic hygiene measures. Relapses, however, occur frequently; particularly in larger families and groups. Treatment with oral ivermectin has proven to remarkably decrease the prevalence of scabies in larger communities with a good tolerability and efficacy. Recent data is additionally hinting toward the safe and effective use of ivermectin in infants weighing <15 kg (6, 15). Besides these two most commonly used treatments in Europe and the US, various other options are available (16). Sulfur compounds are widely used in Afrika and South America due to good efficacy, however, skin irritations are frequent. Benzyl benzoate is highly antiscabietic but also bares high rates of skin irritation. Both treatment options are commonly used in poorer regions due to their efficacy and low cost. Crotamiton is a topical treatment that shows a good tolerability and is therefore used in children with unsatisfactory success (16).

## Clinical Characteristics of Itch in Patients Infected With Scabies

The infestation with scabies poses a high burden on respective patients, often due to an intense unbearable itch. The symptom lasts as long as scabies lasts, however, it might become chronic due to persistence after therapy of the infestation. Thus, scabies should be excluded in cases of persisting pruritus especially with a history of itch in persons with close skin contact (17). In severe cases, the patients even develop papules due to scratching (prurigo), or eczema. Management of chronic itch is often challenging (18) and especially scabies-specific itch is poorly characterized and understood. Itch can deteriorate over the night (nocturnal crescendo). This, however, is not specifically applicable to scabies, as other common skin diseases also display a nighty worsening of itch, that is, atopic dermatitis and psoriasis (19–22). Concerning other clinical symptoms, Brenaut et al. found that heat sensations accompanying the itch were significantly less frequent as compared to the other pruritic skin diseases (19). Sweating and hot water increased the intensity of itch in 73 and 67% of scabies patients, respectively. Scratching was considered pleasurable in 47% of scabies patients as compared to 69 and 65% of patients with atopic dermatitis and psoriasis, respectively. Interestingly, while scratching lesions in patients infected with scabies (63% of patients, *p* < 0.01) were significantly more frequent as compared to non-atopic eczema, psoriasis and urticaria, patients with scabies revealed the lowest rate of lichenification (8%) compared to 80, 61, and 33% of patients with atopic dermatitis, eczema and psoriasis, respectively. Of note, one major limitation of the study is the depiction of qualitative but not quantitative itch features between the aforementioned dermatosis (19).

The presence of pruritus is reported in most of the patients infected with scabies and the prevalence ranges from 90–99% in current literature (23). Nair et al. found a reported prevalence of pruritus in 99% of 102 adults and a nocturnal aggravation in nearly 80% of the patients leading to sleep disturbances in a prospective, observational cross-sectional study conducted at a tertiary center (24). Similar data regarding the manifestation of itch was reported from a cohort of 323 pediatric patients (25). Here, the overall itch prevalence accounted for 94.5% with a range from 90.3–96.9%. Interestingly, the authors showed that the sensation of itch increases with the age of the pediatric patients characterized in the cohort (25). Itch, however, is more difficult to assess in infant patients and is often displayed as discomfort, crying and an increased irritability what might explain the aforementioned observation.

Comparing classical scabies with scabies crustosa/norvegica, differences in pruritus have been reported, indicating that the itch intensity is lower in patients with crusted scabies. However, still most of the patients with crusted scabies had pruritus to some extend (10, 11). Interestingly, in the crusted scabies cohort characterized by Roberts et al. more than half of the patients bared an identifiable immunosuppressive risk. The authors stated that in patients without respective risks, the development of the crusted clinical appearance might result from an increased tendency to mount a Th2 immune response (10). Furthermore, crusted scabies is reported to affect predominantly individuals with malnutrition, Down's syndrome, the elderly and patients with deficient cognitive abilities or physical debilities who are unable to appropriately depict and to react to itch by scratching (10, 26, 27).

## Pathophysiology of Itch in Scabies

Yet, insights into the exact underlying pathomechanisms of itch in scabies remain scant while remarkable progress in understanding itch in principal had been made in the past 20 years (23). The pathophysiology of itch in general includes the direct stimulation of itch-sensory neurons in the skin by epithelial-cell-derived cytokines, that is, IL-33 and thymic stromal lymphopoietin (TSLP) and an indirect stimulation of itch by keratinocyte-derived kalikreins (KLK) like KLK7. Furthermore, the effector cytokines IL-4, IL-9, IL-13, and IL-31 and CXCL10 directly promote itch (28). Regarding the pruritogens IL-4, IL-13, and downstream JAK activation, no studies investigated this in scabies in detail.

However, hypothesis can be derived from the immunologic reaction ongoing in a patient infected with scabies and by using a novel porcine animal model for scabies, thus, deeper insights into the scabies-specific itch can be generated.

The major immune response to the mite infestation includes the innate immune system and the activation of the complement system that, so some extent, can be inhibited by components produced by the mites. Effectors of the immune response include activated mast cells, immunoglobulin E (IgE), eosinophils, and non-histaminergic effectors like PAR2 and IL-31. While in the classical non-crusted scabies, a Th1 mediated immune response plays a predominant role, a Th2 immune response seems to be more important in the pathogenesis of crusted scabies (29).

Albeit immunohistochemical analysis, using a basophil-specific BB1 antibody, revealed numerous basophils infiltrating lesional scabies-infested skin, the pathogenetic significance of this observation remains unclear and requires further investigations (30, 31).

Current hypothesis regarding the pathophysiology of itch in scabies can be subdivided into the direct action of the scabies mite and the immune response toward the mite itself (23).

Mite components can directly lead to an activation of the Toll-like receptor (TLR) pathway with a subsequent activation of TLR 3, 4, and 7 that are expressed on primary sensory neurons (32, 33). Mite feces contain proteases that can lead to an activation of protease-activated receptor 2 (34). The close interaction between the mites and the keratinocytes can lead to the release of protease activating protease-activated prurireceptors (23, 35). Furthermore, mite components that can be recognized as antigens and show a similar structure to antigens of the house dust mite, can induce an IgE-mediated mast cell activation with an aggravation of itch mediated by the degranulation of histamine, tumor necrosis factor (TNF) alpha and tryptase with a subsequent activation of histaminergic H1 and H4 prurireceptors and protease-activated prurireceptors by tryptase (36, 37). The itch sensation can further be enhanced by a release of leukotriens and prostaglandins implicated my macrophages (23).

In the Th1-mediated immune response that is primarily present in classical non-crusted scabies, a release of INF-gamma and interleukin(IL)-2 lead to an activation of cytokine prurireceptors, whereas an up-regulation of the Th2-mediated immune response in crusted scabies elevates eosinophils, IgE-activated mast cells and enhances the activation of cytokine prurireceptors by IL-31 (23, 37). Recently, it was shown that increased IL-31 levels from murine peritoneal macrophages were induced by an overexpression of thymic stromal lymphopoietin and periostin in an experimental mouse model (38). However, the relevance of this non-histaminergic pathway in the scabies itch is yet to be further investigated as compared to the histaminergic pathway.

In the recent years, a porcine model for scabies was established facilitating the investigation of specific scabies-related questions (39–41). Recently, Sanders et al. investigated the potential mechanisms of scabies itch and found that non-histaminergic mediators of pruritus were significantly elevated in the skin of pigs experimentally infested with scabies as well as in human skin as compared to non-infected healthy controls (42). Accordingly, a significant upregulation of TRPV1, TRPA1, and PAR-2 expression in the epidermis and an increase of tryptase+ cells around the dermal-epidermal junction was found in both porcine and human scabies-infected skin. These data suggest that the non-histaminergic mediators might play an important role in scabies itch and might potentially serve as therapeutic targets. Furthermore, the similar results from human and pig skin biopsies indicate that the porcine model might serve as suitable animal model to investigate the scabies-specific itch in future experiments. Slight variances in the data might result from different biopsy sites, different durations of the disease, scratching and general differences between the species (42).

## Complications Linked to Itch in Scabies

Usually, secondary complications and morbidity of individuals infected with scabies are discussed as a direct consequence of the infestation with scabies. However, it is more accurate to refer to subsequent complications due to massive itch caused by an immune response toward the mites and the direct interaction of complement inhibitors produced by the mites and complement pathways in the skin enabling bacteria to grow more easily. Furthermore, severe itch and subsequent scratching leads to a disruption of the epidermal skin barrier (43) and thus to a skin more susceptible for bacterial skin infections. Due to the ability of the scabies mite to interfere with the human complement system by blocking all three complement initiation pathways and leading to decreased neutrophil functions, staphylococcal and streptococcal growth is promoted (44–50). A study performed using the porcine animal model provided evidence that the skin microbiome is changed due to the scabies infestation enabling the growth of opportunistic pathogens (51). In literature, impetigo is described as the most frequent complication of scabies-related itch. It commonly affects children and individuals living under crowded conditions in tropical regions (4, 52, 53). Excoriated deep skin lesions harboring bacteria, most frequently Staphylococcus aureus and group A Streprococcus (AGU), can further lead to a hematogenic dissemination with subsequent complications involving other organs. Interestingly, scratching lesions appear more frequently in patients infested with scabies as compared to other itching dermatosis i.e. eczema, psoriasis and uritcaria (19). The bacterial superinfection can lead to local infections (i.e., erysipelas, cellulitis, abscesses, staphyloderma) with/or without systemic affection and in the worst case to a sepsis. Post-streptococcal complications can affect the kidneys (glomerulonephritis), the heart (rheumatic heart disease) and the joints (acute rheumatic fever) and pose a high burden on respective patients and the healthcare system (54–57). Patients infected with scabies, thus, suffer from an impaired life quality that is directly linked to the severity of itch.

## Conclusion

Scabies is a common and neglected skin infestation characterized by severe itch and a heterogenous clinical picture. Itch in scabies can be caused by direct mite actions and by a resulting immune response toward the mites. Recent data from the porcine animal model hint toward an important role of non-histaminergic itch mediators, that is, TRPV1, TRPA1, PAR-2, and tryptase+ cells. The exact pathomechanism of scabies-specific itch remains yet to be further investigated.

## Author Contributions

All authors contributed to the article and approved the submitted version.

## Conflict of Interest

The authors declare that the research was conducted in the absence of any commercial or financial relationships that could be construed as a potential conflict of interest.
